# Complete Genome Sequence of the Cytokinin-Producing Biocontrol Strain Pseudomonas fluorescens G20-18

**DOI:** 10.1128/MRA.00601-21

**Published:** 2021-07-29

**Authors:** Tue K. Nielsen, Mengistu F. Mekureyaw, Lars H. Hansen, Mette H. Nicolaisen, Thomas G. Roitsch, Rosanna C. Hennessy

**Affiliations:** a Department of Plant and Environmental Sciences, University of Copenhagen, Frederiksberg, Denmark; Indiana University, Bloomington

## Abstract

Here, we report the complete genome sequence of the cytokinin-producing plant growth-promoting strain Pseudomonas fluorescens G20-18. The complete genome assembly resulted in a single, circular chromosome of 6.48 Mbp and harbors several secondary metabolite biosynthesis gene clusters that are potentially involved in its plant growth-promoting function.

## ANNOUNCEMENT

Pseudomonas fluorescens strain G20-18 is a plant beneficial bacterium that produces the phytohormone cytokinin, which activates plant resistance and prevents disease (1–3). P. fluorescens G20-18 was previously isolated from Arctic grass (Ellesmere Island, Canada) using an assay designed to select for bacteria that promote soybean seedling emergence from cold soil. The original isolation of strain G20-18 was performed on Pseudomonas agar F at 14°C for 2 weeks (4). Genomic DNA for Illumina and Nanopore sequencing was purified from a single colony grown at 28°C in lysogeny broth (LB) using the Gentra Puregene yeast/bact. kit (Qiagen, Germany), according to the manufacturer’s instructions. Library preparation for Illumina sequencing was performed using the Nextera XT DNA library preparation kit (Illumina, Inc., San Diego, CA, USA). Sequencing was carried out on an Illumina NextSeq 550 instrument using the midoutput kit v2.5 (300 cycles) (Illumina, Inc.). Adapter sequences were trimmed from Illumina reads using Trim Galore (v0.6.4) (5) with default settings. For Nanopore sequencing, a library was prepared using the rapid barcoding sequencing kit (SQK-RBK004) which was sequenced on a MinION Mk1B device using an R9.4 flow cell. Raw Nanopore reads were base called with Guppy (v4.4.1), trimmed for adapter sequences with Porechop (v0.2.4), and assembled with Flye (v2.8.3) (6). The assembled genome was polished with trimmed Illumina and Nanopore reads using the unicycler_polish module from the Unicycler assembler (v0.4.8) (7), which applies multiple rounds of polishing with Pilon (v1.23) (8) and Racon (v1.4.3) (9). Genome annotation was conducted using Prokka (v1.14.6) (10). For the publicly available genome of G20-18, annotation was performed using the NCBI Prokaryotic Genome Annotation Pipeline (PGAP) v5.2 (11).

A total of 1,154,966 read pairs were obtained from Illumina sequencing and 78,118 reads from Nanopore sequencing with an *N*_50_ read length of 12.2 kbp and a total of 528 Mbp.

The complete genome sequence of P. fluorescens G20-18 is 6,478,110 bp with a mean G+C of 59.09%. The Prokka annotation of the G20-18 genome revealed the presence of 5,917 predicted coding sequences (CDS) and 93 predicted RNA (19 rRNAs, 72 tRNAs, and 2 transfer-messenger RNAs [tmRNAs]) coding sequences. Several potentially relevant secondary metabolite biosynthetic gene clusters were identified using antiSMASH 6.0 (12), including the lankacidin C, fengycin, pyoverdine, l-2-amino-4-methoxy-trans-3-butenoic acid, and arylpolyene biosynthesis clusters that may help to explain the plant beneficial role of strain G20-18, which will be further explored in future studies. The presence of the previously described cytokinin biosynthetic gene tRNA delta(2)-isopentenylpyrophosphate transferase (*miaA*) (1) was confirmed.

### Data availability.

The whole-genome shotgun project has been deposited in GenBank under the accession no. CP075566. Raw sequence reads are available under SRA accession no. SRR14640385 (Nanopore) and SRR14640384 (Illumina).
